# Protein Carbamylation: A Marker Reflecting Increased Age-Related Cell Oxidation

**DOI:** 10.3390/ijms19051495

**Published:** 2018-05-17

**Authors:** Julia Carracedo, Rafael Ramírez-Carracedo, Irene Martínez de Toda, Carmen Vida, Matilde Alique, Mónica De la Fuente, Rafael Ramírez-Chamond

**Affiliations:** 1 Department of Genetics, Physiology, and Microbiology, Faculty of Biology, Complutense University/Instituto de Investigación Sanitaria Hospital 12 de Octubre (imas12), 28040 Madrid, Spain; julcar01@ucm.es (J.C.); irene_mc90@hotmail.com (I.M.d.T.); carmenvidarueda@hotmail.com (C.V.); mondelaf@bio.ucm.es (M.D.l.F.); 2 Cardiovascular Joint Research Unit, Francisco de Vitoria University/Hospital Ramon y Cajal Research Unit (IRYCIS), 28223 Madrid, Spain; rrcarracedo@hotmail.com; 3 Biology Systems Department, Physiology, Alcala University, Alcala de Henares, 28805 Madrid, Spain; matilde.alique@uah.es

**Keywords:** aging biomarker, functional immune signature, immune profile, lipid peroxidation, malondialdehyde, oxidative damage, protein carbamylation

## Abstract

Carbamylation is a post-translational modification of proteins that may partake in the oxidative stress-associated cell damage, and its increment has been recently proposed as a “hallmark of aging”. The molecular mechanisms associated with aging are related to an increased release of free radicals. We have studied whether carbamylated proteins from the peripheral blood of healthy subjects are related to oxidative damage and aging, taking into account the gender and the immune profile of the subjects. The study was performed in healthy human volunteers. The detection of protein carbamylation and malondialdehyde (MDA) levels was evaluated using commercial kits. The immune profile was calculated using parameters of immune cell function. The results show that the individuals from the elderly group (60–79 years old) have increased carbamylated protein and MDA levels. When considered by gender, only men between 60 and 79 years old showed significantly increased carbamylated proteins and MDA levels. When those subjects were classified by their immune profile, the carbamylated protein levels were higher in those with an older immune profile. In conclusion, the carbamylation of proteins in peripheral blood is related to age-associated oxidative damage and to an aging functional immunological signature. Our results suggest that carbamylated proteins may play an important role at the cellular level in the aging process.

## 1. Introduction

Increased non-enzymatic post-translational modifications of plasmatic proteins are a common finding in the elderly, and their quantification has been used as an aging biomarker [1]. Modifications of proteins can be the consequence of several metabolic processes and are often induced by an unbalanced cellular function or by inflammatory and oxidative factors. Under physiological conditions, these modified proteins are usually degraded since they may induce altered signals in cells, which stimulates pathological responses [2]. However, in elderly people, these modified proteins accumulate in peripheral blood, likely due to physio-pathological situations, which can modify their synthesis and degradation. Increased oxidative stress associated with the aging process usually induces post-translational modifications of proteins including glycation, glycoxidation, lipoxidation and carbonylation [3] (reviewed in Korovila et al., 2017). This can modify their synthesis and degradation [4]. Consequently, modified proteins in the elderly may develop damage and pathologic processes. In addition, increasing evidence supports the main role for these modified proteins among others in the aging process of cardiovascular diseases [5].

Among the age-related modifications of proteins, carbamylation has been proposed as a post-translational protein modification that may participate in the cellular damage associated with oxidative stress [6]. Protein carbamylation is produced by the binding of isocyanic acid to free amino groups of proteins and, preferentially, to the fragments ε-NH2 of lysine residues, which generates homocitrulline. Isocyanic acid comes mainly from the dissociation of urea into cyanate and ammonium, which explains the elevated levels of carbamylated proteins in renal pathology [7,8]. It is also found in the atmosphere because of pyrolysis/combustion of biomass [9]. Plasma concentration of myeloperoxidase (MPO) increases in the elderly and/or patients with several aging-related inflammatory diseases such as cardiovascular diseases, type II diabetes or chronic kidney disease. The action of the enzyme myeloperoxidase on thiocyanate generates isocyanic acid [10,11,12,13,14,15]. Protein carbamylation has risen as a particular point of interest in recent years since there is some clinical and experimental evidence of the association between serum [16] carbamylated protein levels and the development of atherosclerosis and cardiovascular diseases [16]. In addition, the carbamylation of proteins may be a key mechanism for triggering autoimmune and inflammatory responses, and it provides a novel mechanism for the pathogenesis of autoimmune or allergic diseases [17,18].

It has been hypothesized that the underlying molecular mechanisms of aging are related to the increased release of free radicals [19,20,21]. The relationship between aging, oxidative stress and subclinical inflammation (oxi-inflamm-aging) explains this process as a progressive accumulation of damaged macromolecules within the older cells. Increased production of reactive oxygen species (ROS) mostly leads to this process [21]. Oxidative stress is defined as an imbalance between the concentration of released oxidants and the activity of antioxidant processes in the organism, which favors the oxidants [22]. Consequently, oxidative stress promotes the oxidation of several molecules such as DNA, lipids and proteins. These resultant oxidized molecules, i.e., albumin [23] or low-density lipoproteins [24], are associated with different processes including aging [25]. During the lipid peroxidation process, polyunsaturated fatty acids attached to the cell membrane are peroxidized by reactions mediated by free radicals. Malondialdehyde (MDA) is the principal end-product in the lipid peroxidation process. It has a long lifespan and high reactivity, which allows it to interact with proteins [26]. The increase of MDA in cells has been related to different processes that can cause tissue damage in the case of inflammation, cancer and aging [26,27,28]. Additionally, it is used as a marker of oxidative stress-associated damage [26].

Human life expectancy is increasing in most countries due to social and medical progress. The primary objective is to reach old age in a healthy state, which favors a higher quality of life and a reduced health cost caused by aging-associated diseases [29]. Actually, it is considered that alternatively to chronological age, there is a biological age determined by the speed at which the cells, tissues and organs deteriorate. This is related to an oxi-inflamm-aging situation [21,30,31]. Along with MDA, some biomarkers are modulated as a consequence of aging-associated oxidative damage and can be useful for determining biological age [32,33]. As such, proper functioning of the immune system can be an excellent health marker [21,34]. In the patients included in this study, several parameters of the immune profile associated with aging have been identified such as chemotaxis, phagocytosis, natural killer (NK) activity and lymphoproliferation in neutrophils and peripheral blood lymphocytes [35]. Those parameters proved to be suitable and useful markers of the aging rate and may be possible markers for determining biological age [35]. Nevertheless, discovering more sensitive and easy to analyze biomarkers is necessary for establishing the health state of a subject. In this context, carbamylated proteins may be potential biomarkers of the age-associated oxidative damage. In addition, the possible differences that may occur between sexes in these markers can be advantageous when making a diagnosis of optimal health status. A few studies in humans have considered the importance of these possible differences.

We aimed to determine whether plasma protein carbamylation is a marker of aging. To achieve this, we first analyzed levels of carbamylated proteins in the process of age-associated natural senescence. Next, we established whether those changes occurred differently in men and women. Finally, we assessed whether the changes in carbamylated protein levels were associated in some way with oxidative damage.

## 2. Results

### 2.1. Protein Carbamylation Is Increased in Plasma from the Elderly

**Figure 1:**
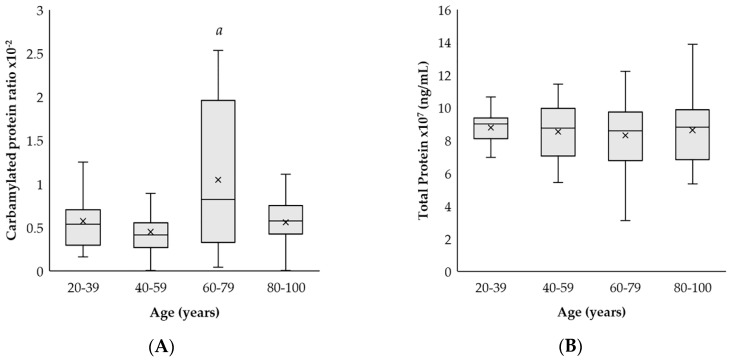
Quantification of carbamylated and total proteins in plasma. Age-related changes in (**A**) the ratio of carbamylated proteins over total proteins and (**B**) the concentration (ng/mL) of total proteins in peripheral blood in 39 adults (20–39 years old), 38 mature (40–59 years old), 24 elderlies (60–79 years old) and 36 long-lived (80–100 years old) human subjects. Each value was made in duplicate. The SD has been calculated from the average. *p* < 0.01 with respect to the value in the other groups.

The carbamylated protein ratio was compared in relation to the age of the subjects. There was a significant increase ratio in the group between 60 and 79 years old (elderly group) when compared to the adult group (20–39 years old) and mature individuals (40–59 years old) (*p* < 0.01). When we analyzed the oldest group (80–100 years; long-lived subjects), we observed a decreased carbamylated protein ratio when compared to the elderly group (60–79 years; *p* < 0.01) (see Figure 1A). The total plasmatic proteins quantification showed no significant differences between the different age groups (see Figure 1B).

### 2.2. Carbamylated Proteins in Male Plasma Are Increased in the Elderly

**Figure 2:**
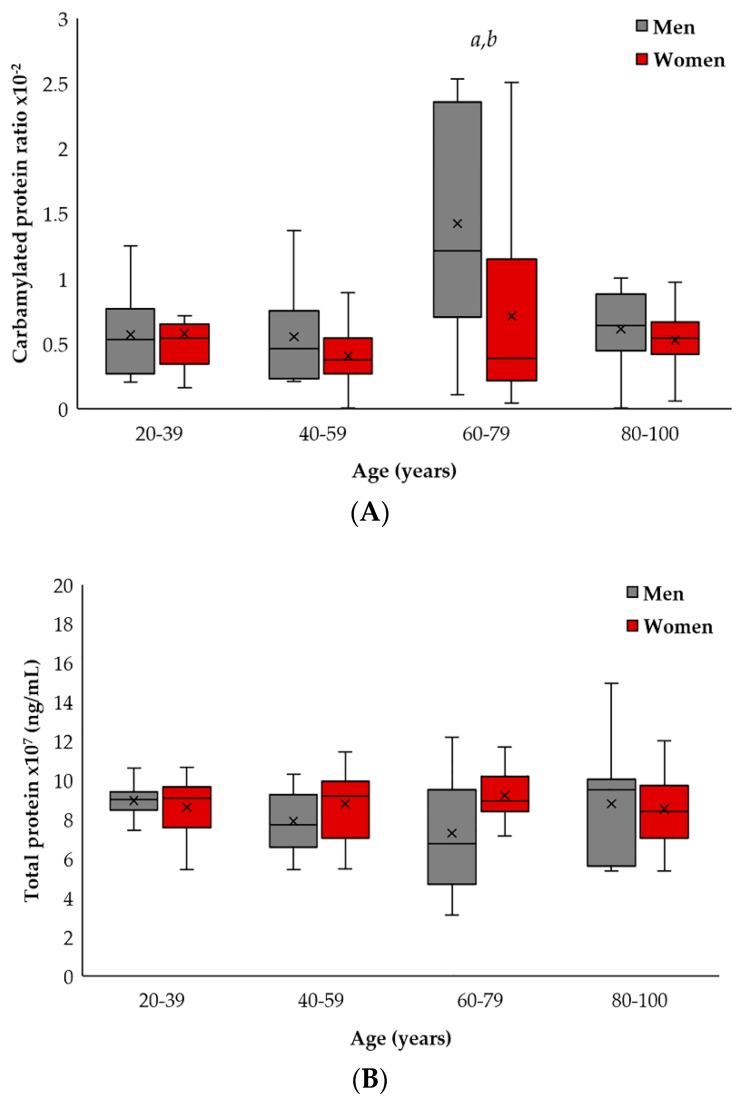
Quantification of carbamylated and total proteins in plasma in relation to age and gender. (**A**) Differences in the carbamylated protein ratio between sexes in relation to age. Changes in the ratio of carbamylated proteins (expressed as carbamylated proteins/total protein) were identified in plasma from men (grey color) (*a*, *p* < 0.01). There were no statistical differences in the carbamylated protein ratio between the women groups (red color). Differences between men and women groups (*b*, *p* < 0.05). (**B**) Concentration (ng/mL) of total proteins in peripheral blood from men and women. Each value was made in duplicate. The SD has been calculated from the average.

In this study, the effect of gender on the carbamylated protein ratio was different among the groups (see Figure 2A), while total protein concentrations showed no difference when the study subjects were broken down by gender (see Figure 2B). Those differences were mostly observed in the elderly group (60–79 years old), where male carbamylated proteins were higher than women’s carbamylated proteins (*p* < 0.05) (see Figure 2). However, the long-lived subjects (80–100 years old) showed no significant differences between men and women, but the carbamylated protein ratio in both sexes was decreased when compared to the previous group (*p* < 0.05).

When analyzed separately, both individual adult and mature men (20–39 years old and 40–59 years old groups, respectively) and the long-lived men (80–100 years old group) showed no differences in the carbamylated protein ratio, while for the above-mentioned individual elderly male group (60–79 years old), it was increased in a significant manner (*p* < 0.05).

Nevertheless, when we compared the separate groups of women, we saw no significant differences even though there was a slight increase in the group of 60–79-year-old individuals. Once again, the 80–100-year-old group of women showed equivalent results to the mature women group (40–59 years old).

### 2.3. MDA Levels in Whole Blood Cells Are Increased in the Elderly

MDA was used as a marker of oxidative stress-associated damage. MDA levels were measured in whole blood cells (erythrocytes and total leukocytes).

**Figure 3:**
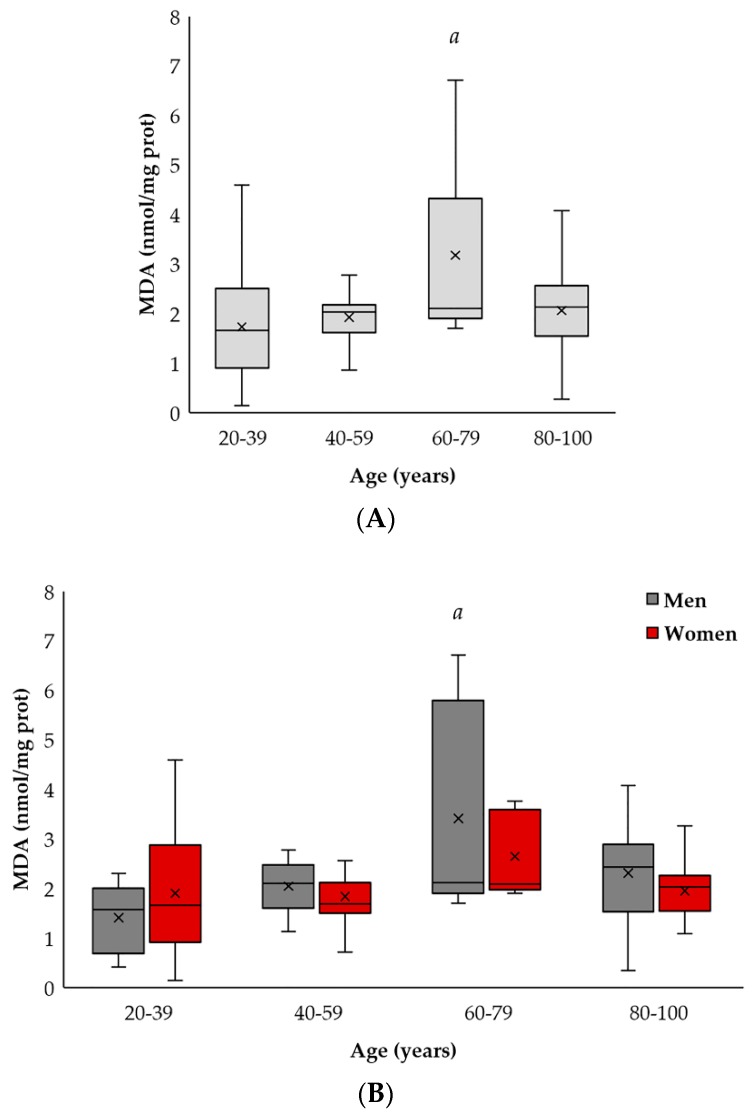
MDA levels (nmol/mg protein) in relation to age and gender were identified in peripheral blood cells. (**A**) Differences between 23 adults (20–39 years old), 24 mature (40–59 years old), 16 elderlies (60–79 years old) and 28 long-lived (80–100 years old) subjects (*a*, *p* < 0.05). (**B**) Differences in the MDA from men (grey color) (*a*, *p* < 0.01). No statistical differences in the carbamylated protein ratio between the women groups (red color) were found. Differences between men and women groups (*b*, *p* < 0.05). Each value was made in duplicate. The SD has been calculated from the average.

As shown in Figure 3A, elderly individuals (60–79 years old) showed higher MDA levels compared to adult (20–39 years old) and mature (40–59 years old) individuals (*p* < 0.05). By contrast, the most elderly subjects (80–100 years old) showed similar levels to those observed in adult and mature individuals.

When subjects were subdivided by gender, it was found that elderly men (60–79 years old) have higher MDA levels than adult ones (20–39 years old) (*p* < 0.05), whereas no statistically-significant age-related differences in MDA levels were found in women (see Figure 3B).

### 2.4. Carbamylation and MDA Levels Are Positively Correlated in Men

**Figure 4:**
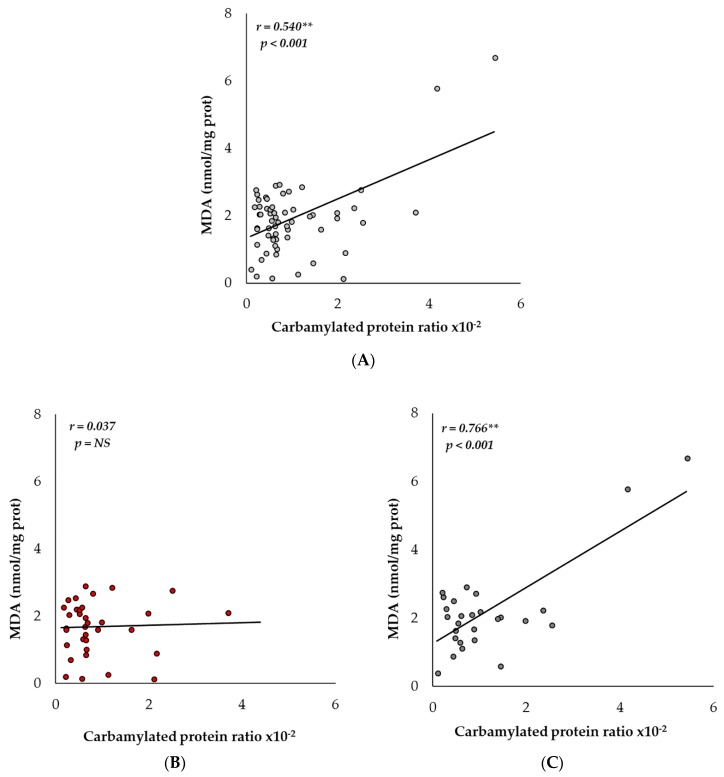
Correlation (Pearson correlation coefficient) between the plasma carbamylated protein/total protein ratio and MDA levels. (**A**) Correlation between all the subjects, men and women, adult (20–39 years old), mature (40–59 years old), elderly (60–79 years old) and long-lived (80–100 years old) subjects (*r* = 0.540; *p* < 0.001). (**B**) No significant (NS) correlation between both parameters in the women group were found. (**C**) Correlation between men, *r* = 0.766; *p* < 0.001). All subjects: light grey dots; women: red dots; men: dark grey dots. ** *p* <0.001.

Once we determined the similar tendencies in the age-associated levels of the carbamylated protein ratio and MDA, we sought to determine whether they were related. When we compared all the grouped subjects of the study, we observed a positive Pearson correlation (*r* = 0.540; *p* < 0.001) (see Figure 4A). As we did before, separate analyses for men and women were performed, and we saw different results. While the levels of women showed no correlation (see Figure 4B), the carbamylated protein ratio and MDA in men was strongly correlated (*r* = 0.766; *p* < 0.001) (see Figure 4C). As we showed previously, the most significant changes happened in the group of men from 60–79 years old (elderly subjects). Therefore, we analyzed that group separately. Even though the number of subjects was fairly low (nine men), we saw a notable correlation between the carbamylated protein ratio and MDA (*r* = 0.910; *p* < 0.001) (see Table 1).

### 2.5. Protein Carbamylation Ratio Is Related to the Immune Profile Associated with Aging

**Figure 5:**
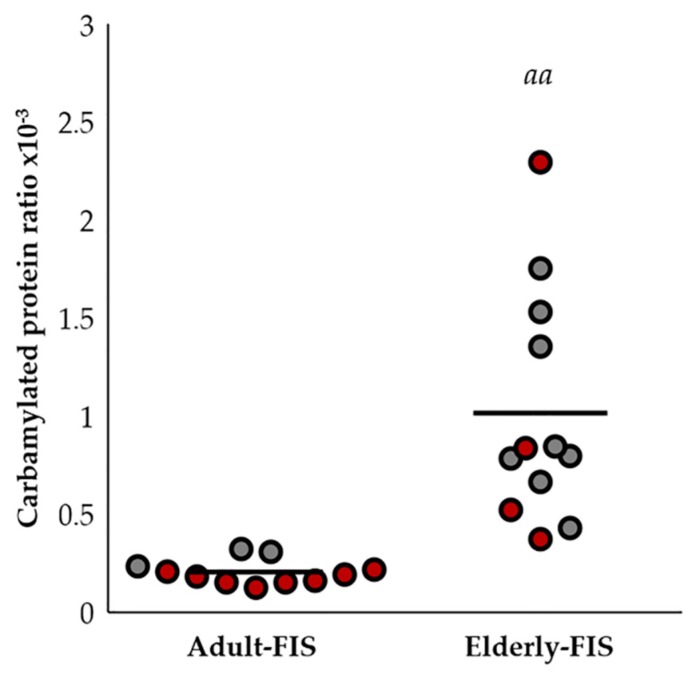
Differences in the carbamylated protein ratio in relation to the functional immune signature (FIS). Changes in the carbamylated protein ratio in relation to FIS (*aa*, *p* < 0.01). Men: dark grey dots; women: red dots.

In a previously-published study [29], these same subjects were analyzed in their immune profile, which was proposed as an indicator of biological age. This immune profile, which was named the functional immune signature (FIS), has been used to classify the subjects in adult-FIS or elderly-FIS. Yet, this depends on their differences in the immune profile. The carbamylated protein levels were significantly higher in the subjects with an aging FIS (see Figure 5). It is important to note that most of the women from this age group showed a lower FIS than the men from the same age group. In relation to the different immune functions analyzed individually, only statistically-significant differences have been found between the carbamylated ratio and natural killer activity (*r* = 0.524; *p* < 0.05).

## 3. Discussion

The search for biomarkers to identify subjects at risk of suffering age-related diseases is a priority to achieve a longer lifespan along with an excellent state of health. Therefore, we studied whether the carbamylated proteins in the peripheric blood of healthy subjects are related to oxidative damage and aging. Moreover, based on our knowledge, this is the first work where the protein carbamylation has been related to the gender of the subjects.

The determination of plasmatic carbamylated proteins confirmed our hypothesis partially. We noticed that the concentration of carbamylated proteins was not linear. The increase was stable until the 60-year-old group had a substantial rise, and later, it was decreased in long-lived subjects (80–100 years old). We sought then to determine carbamylated albumin since it is the most common plasmatic protein. Some groups have shown a decrease of albumin concentration with aging [36,37], while others have demonstrated that age is not a cause of hypoalbuminemia [38]. The latter studies declare that hypoalbuminemia is more related to the state of health in older subjects [39,40], which is a prognostic factor of mortality in the elderly. Our results confirm this hypothesis since the total levels of albumin only showed a few insignificant differences between the groups. First, our results confirmed that in non-pathologic subjects, the total albumin is not modified at all. Therefore, the differences between the carbamylated proteins were not due to changes in the total amount of protein. Our work shows that the variations observed are not caused by total protein modifications, but are due to carbamylated protein level changes.

At this point, we wanted to know the biological meaning of those changes. Other studies suggest that an increase in carbamylated protein levels contributes to aging [16], and carbamylated protein levels have been recently proposed as the “hallmark of aging” in mammalian skin tissues [1]. Nevertheless, to date, this is the first time that this fact has been demonstrated in a study with healthy subjects from a wide range of age including a long-lived group.

Recently, it was published that gender modifies the serum albumin levels in relation to age [41]. Alternatively, we raised the possibility that we were making a mistake by studying the data from men and women together. For this reason, we evaluated the data separately, and this new approach showed us better results. Although women showed an increasing tendency in the number of carbamylated proteins in the group of 60–79 years old, this difference was not significant when compared to the other groups. By contrast, the male group of 60–79 years old presented substantial differences, and those results were not related to the total number of proteins.

Even though we were aware that the small number of subjects could condition the interpretation of the results, we focused on the group from 60–79-year-olds. The observed dispersion suggested that this group contained subjects with and without age-related oxidative stress. The correlation study showed that the subjects with low or moderate oxidative stress levels also had low or moderate carbamylated protein levels. We suggest that those results may explain, at least in part, the differences between sexes in the tendency to suffer some pathologies such as cardiovascular diseases [42,43]. Recently, it has been published that the carbamylation of low-density lipoproteins (LDL) induces endothelial dysfunction through increased production of ROS and the uncoupling of endothelial nitric oxide synthase (eNOS) [44]. Further studies are needed to delve into the implied mechanisms, since greater knowledge can lead to better options at the clinical level, particularly in men. Therefore, harmful processes in the endothelium and the development of cardiovascular pathologies can be prevented.

At this point, we considered that it might not be the chronologic age that was conditioning the carbamylated serum protein levels, but age-related damage. To study the age-related oxidative damage, we chose MDA as a marker of imbalanced oxidative stress. MDA has been identified as a fairly accurate marker of oxidative stress [45]. The use of MDA as a biomarker is limited due to the technical difficulties of determining it [46]. MDA is a consequence of lipid modifications by ROS and is considered the most mutagenic product of lipid peroxidation. MDA is an end-product generated after the decomposition of arachidonic acid and larger polyunsaturated fatty acids, which are the major substrates in lipid peroxidation [47], through enzymatic and non-enzymatic processes. MDA is also one of the most popular markers for determining oxidative stress in clinical situations [48]. Because of its toxicity, MDA has been used for years as a biomarker in the lipid peroxidation of fatty acids due to its reactivity with thiobarbituric acid reactive substances (TBARS) [49]. However, the reactions with TBARS are not notably specific, which leads to controversies about its use in vivo. Alternatively, some technologies for determining free and total MDA such as gas chromatography-mass spectrometry (GC-MS/MS), liquid chromatography-mass spectrometry (LC-MS/MS), and several derivatization-based strategies have been developed during the last decade [46,47,48,49]. The obtained results were similar to those from the carbamylated proteins, since it rose markedly in men, but there were no significant changes in women. Concretely, this marker was intensely increased in men from 60–79 years old to return to basal levels from this age. Similar to us, Rizvi and Maurya, 2007 [50], have shown an age-dependent increase in erythrocyte MDA levels in 80 normal healthy volunteers that correlated significantly with a total antioxidant capacity of the plasma [50]. When we related the levels of stress and carbamylated serum albumin, we obtained a *p*-value less than 0.001. Therefore, this result shows that the carbamylated serum albumin levels might not reflect the chronologic age of the subjects, but age and sex-associated damage were also changed between genders.

Some authors suggested that chronological age may not be the most accurate indicator of aging or the state of health, but the biological age is the most appropriate way to measure the rate of aging [16]. In this sense, several studies suggested that a large number of markers is needed to estimate the biological age of a subject accurately [51]. Recently, some immune function parameters have been identified and validated as markers of biological age. Studying the same subjects in this work, Martinez de Toda et al., [35] analyzed parameters of immune function. Their work exposed that elderly people (60–79 years old) showed a decrease in all the previous functions. In a similar way to our study, the long-lived subjects (80–100 years old) maintained identical values to the adults (20–39 years old). Therefore, our results are parallel to those by adding carbamylated proteins as an additional biomarker of aging associated with the FIS. Therefore, our study establishes a new parameter to determine the biological age of a subject. The benefit of using carbamylated proteins lies in the ease of quantifying them. Since the previous results from the immunologic function in humans were confirmed in a murine model [35,52], our results must be confirmed in an animal model.

Additionally, the concentration of carbamylated proteins in long-lived subjects (80–100 years old) decreases to levels comparable to those of adults (20–39 years old). This lesser amount of carbamylated proteins has been related to reduced oxidative damage. In animal models that reach a long lifespan [52], the proliferative responses of lymphocytes in a basal state remain low. However, these cells show better responses when stimulated. Notably, long-lived mice showed an even higher basal release of pro-inflammatory cytokines such as IL-1β and IL-6 when compared to their old age. It is thought that appropriate modulation of the immune response and cytokine release in long-lived mice could explain their high resistance to infections and their high longevity. Those results can guide us to comprehend the data obtained in this study. We agree with other authors that both humans and animals with prolonged lifespans have adapted successfully to adverse situations [53,54]. For example, it is known that low-grade chronic inflammation is common in the elderly (e.g., 79 years old) and centenarian individuals. However, there are differences regarding the anti-inflammatory agents between elderly subjects since those who reach exceptionally long lives can avoid the main age-related diseases [55,56]. It is important to note that long-lived subjects with a low biological age will have a higher life expectancy, and they will have a better state of health. Consequently, identifying the diverse factors modified during aging will help us discover new biomarkers and new targets to act in a preventive and therapeutic manner. There are some factors that are not registered in this study and can improve the stress levels by increasing the antioxidants. For example, caloric restriction is known to have a better redox state [57,58,59]. In addition, exercise can also be an important part of a healthy lifestyle [60,61]. Studies designed to consider those factors should be conducted in the future.

Therefore, the carbamylated proteins ratio is a biomarker that can be a potential indicator of subjects at risk of suffering associated diseases. Furthermore, we consider that the notable decrease of both parameters from the 79-year-old group may indicate that those subjects have overcome this risk stage. This statement needs to be confirmed in the future by increasing the number of subjects in the study, but it opens an interesting perspective since it is a group that used to be excluded in all the studies of aging and disease. In the future, a longitudinal study in mouse models, which is more viable than humans, could increase knowledge about the value of these studies in aging since it has been shown that the results are comparable.

## 4. Materials and Methods

### 4.1. Study Subjects

In total, 137 human volunteers participated in this study (86 women; 51 men). The volunteers’ age range was comprised of 20–100 years old, divided into four different experimental groups: adult (20–39 years old, 24 women and 15 men), mature (40–59 years old, 26 women and 12 men), elderly (60–79 years old, 12 women and 12 men) and long-lived subjects (80–100 years old, 24 women and 12 men).

All the volunteers were in healthy condition (absent of pathology or findings of clinical significance in general laboratory parameters). The criteria for selecting volunteers excluded individuals showing obesity, smoking or alcoholic habits, acute or chronic diseases (such as diabetes mellitus, asthma or cardiovascular disease) or organ dysfunction. In addition, the individuals had not taken any medication. The participants voluntarily authorized the anti-aging physician to use their blood samples for different evaluations. The local Scientific Ethics Committee approved the present research protocol.

### 4.2. Blood Samples

Venous blood samples were drawn from subjects into sodium citrate-buffered Vacutainer tubes (BD Diagnostic, Spain) and coated tubes. Plasma was prepared at room temperature by centrifugation (15 min at 1500× *g*). The supernatant was stored in 1.5-mL tubes at −80 °C until use. By contrast, whole blood cells (erythrocytes and total leukocytes) were obtained following a previously described procedure [46]. For this, 500 µL of the peripheral blood were diluted with 500 µL of RPMI 1640 medium without glutamine (Gibco, Burlington, ON, Canada) and 10 µL gentamicin (0.1 mg/mL in the tube). The samples were incubated for 4 h at 37 °C in a saturated atmosphere of CO_2_ and humidity. Afterward, samples were centrifuged at 900× *g* for 10 min to obtain the whole blood cell pellets after plasma removal. RPMI 1640 with glutamine (Gibco, Canada) was added to the blood cells to make 1 mL. These aliquots were stored at –80 °C until they were used for determining the MDA levels.

### 4.3. Total Protein Quantification

Total protein concentration (ng/mL) in plasma was determined by the Biuret method using a Biuret Assay Kit from BioSystems (Barcelona, Spain). The functional groups in the protein’s peptide bonds bound to Cu^2+^ ions in an alkaline medium, which generated colored complexes. The absorbance (545 nm) is then directly proportional to the amount of total proteins in the sample when compared to an albumin standard curve.

### 4.4. Carbamylated Protein Quantification

The detection and quantification of protein carbamylation were performed using the OxiSelect Protein Carbamylation Sandwich ELISA Kit from Cell Biolabs (San Diego, CA, USA). The samples were analyzed following the manufacturer’s instructions, and the carbamylated protein concentration (ng/mL) was related to the total protein to establish a ratio (carbamylated proteins/total protein), discarding the fact that our results could be due to differences in the total protein between samples.

### 4.5. Lipid Peroxidation (MDA) Assay

Determination of malondialdehyde (MDA) levels was evaluated using the commercial kit “MDA Assay Kit” (Biovision, Milpitas, CA, USA), which measures the reaction of MDA with thiobarbituric acid (TBA) and the MDA-TBA adduct formation. To accomplish this, human whole blood aliquots (100 μL) were resuspended in 200 μL MDA lysis buffer with 2 μL butylated hydroxytoluene (BHT) (0.1 mM), sonicated and then centrifuged at 13,000× *g* for 10 min. The supernatants (200 μL) from each sample were added to 600 μL of TBA and incubated in a water bath at 95 °C for 60 min. Samples were cooled down on ice for 10 min, and 300 μL of n-butanol (Sigma-Aldrich, St. Louis, MO, USA) were added to create an organic phase in which the MDA molecules were to be placed. Samples were centrifuged for 10 min at 13,000× *g* at room temperature and 200 μL of the supernatants (upper organic phase) were collected and dispensed into a 96-well microplate for spectrophotometric measurement at 532 nm. MDA supplied in the kit was used as a standard, and MDA levels were determined by comparing the absorbance of samples with that of the standards. To prevent further formation of MDA during the preparation of the sample or during the heating step, an antioxidant was used. BHT, which is one of the most widely-used compounds, was applied when performing this assay. The protein content of the samples was determined by following the bicinchoninic acid (BCA) protein assay kit protocol (Sigma-Aldrich) and by using serum albumin (BSA, Sigma-Aldrich, USA) as the standard. Results were expressed as nmol of MDA per mg of protein.

### 4.6. Functional Immune Signature

The immune profile was calculated by measuring immune cell function parameters, including chemotaxis, phagocytosis, natural killer activity and lymphoproliferation in neutrophils and lymphocytes isolated from peripheral blood. The parameters in these same patients have been previously published [35].

### 4.7. Statistical Analysis

Analyses were performed with SPSS 21.0 (SPSS, Chicago, IL, USA) software. All data were presented as the means ± standard deviation (SD). The normality of the samples and homogeneity of the variances were checked by the Kolmogorov–Smirnov test and the Levene test, respectively. Differences due to age were studied through the Student *t*-test or one-way analysis of variance (ANOVA) followed by post hoc test analysis. The Tukey test was used for post hoc comparisons when variances were homogeneous, while its counterpart analysis, Games–Howell, was used with unequal variances when they were not homogeneous. Pearson’s correlation coefficient (*r*) was used to test for correlation between the different parameters. Two-sided *p* < 0.05 was considered the minimum level of significance.

## 5. Conclusions

Our data indicate that determining carbamylated plasmatic proteins can be performed with low disturbances for the subject, a low economic cost and by reflexing the age-associated oxidative stress damage. Consequently, and even though more complex studies are required, our results show that the determination of carbamylated proteins may be a useful biomarker to identify subjects at risk of suffering age-related diseases.

## Figures and Tables

**Table 1:** Pearson’s correlation coefficients between the plasma carbamylated protein/total protein ratio and the MDA levels in whole blood cells from elderly subjects (60–79 years old).

Age Group	*r*	*p*-Value
All subjects (*n* = 16)	0.841	0.004
Woman (*n* = 7)	0.008	0.986
Men (*n* = 9)	0.910	0.001

Data are shown as the Pearson’s coefficient (*r*) values corresponding to the number of subjects analyzed in the elderly group (seven women and nine men).

## Abbreviations

MDA	Malondialdehyde
ROS	Reactive oxygen species
eNOS	Endothelial nitric oxide synthase
LDL	Low-density lipoproteins
NK	Natural killer
FIS	Functional immune signature
TBARS	Thiobarbituric acid reactive substances
GC-MS/MS	Gas chromatography-mass spectrometry
LC-MS/MS	Liquid chromatography-mass spectrometry
BHT	Butylated hydroxytoluene
BCA	Bicinchoninic acid
